# Factors associated with scientific production of professors working at a private university in Peru: An analytical cross-sectional study

**DOI:** 10.12688/f1000research.126143.1

**Published:** 2022-10-26

**Authors:** Oriana Rivera-Lozada, ISABEL CRISTINA RIVERA-LOZADA, Cesar Antonio Bonilla-Asalde

**Affiliations:** 1 South American Center for Education and Research in Public Health, Universidad Norbert Wiener, Lima, UNIVERSIDAD NORBERT WIENER, LIMA, Lima 32, Peru; 2 Universidad del Cauca, Popayán, Colombia, Universidad del Cauca, Popayán, Colombia, Popayan, 190002, Colombia; 3 School of Human Medicine, Universidad Privada San Juan Bautista, Chorrillos, Lima, Peru, UNIVERSIDAD PRIVADA SAN JUAN BAUTISTA, LIMA, Lima 32, Peru

**Keywords:** Scientific production, Publication, Professors, Universities, Research Culture

## Abstract

**Objective:** To estimate the association between the academic, personal, and work characteristics and scientific production of professors at a private university of Lima, Peru, in 2021. **Methods:** We undertook an observational, analytical, and cross-sectional study. The sample included 322 professors through simple random sampling. Two questionnaires were administered. The first gathered personal, academic, and work characteristics; while the second evaluated scientific production. The chi-squared test was used, with a significance level of p<0,05, to evaluate the association between the different characteristics and scientific production. A multiple logistic regression was analyzed through the Stepwise method to evaluate the relationship between the variables of exposure and scientific production. We calculated prevalence ratios (PRs) with their respective 95% confidence intervals (95% CI). **Results:** We analyzed 322 professors, 59,6% were male. Scientific production was associated with being registered in Renacyt (PR = 5,52; 95% CI: 2,14 to 4,23; p = <0.001), having a doctoral degree (PR = 2,45; 95% CI: 1,60 to 3,85; p = <0.001), having been a thesis advisor (PR = 3,83; 95% CI: 1,45 to 5,66; p = <0.001), having facilities to conduct research at the workplace (PR = 1,58; 95% CI: 1,12 to 2,47; p = 0.006), and having received training by the university (PR = 1,99; 95% CI: 1,55 to 2,56; p =0.001). **Conclusions:** Scientific production was associated with being registered in Renacyt, having a doctoral degree, having been a thesis advisor, having facilities to conduct research at the workplace, and having being trained in research by the university. Hence, evaluation systems and the monitoring of university quality standards should be strengthened. In addition, it is necessary to undertake wider scope studies in order to enhance the strategies that promote professors’ research.

## Introduction

Scientific production is one of the most important indicators of scientific and technological development. ^1^ It is closely related to the economic and social development of a country or region, ^2^^,^^3^ as it helps researchers disseminate knowledge through the publication of scientific articles in indexed journals. The countries that lead the scientific output rankings are the United States, China, and Great Britain; while in Latin America, Brazil, Mexico, and Argentina are in the top of the lists. ^4^^,^^5^ In Peru, the efforts to reach scientific and technological development through the investment in education and research projects with a great impact are scarce. Thus, this country is in the 73rd place in terms of scientific production at a world level and in the eighth in Latina American. ^5^

In the world, universities constitute the ideal environment for the generation of evidence through scientific research. ^6^ They have a fundamental role in scientific production by promoting and producing new knowledge that help the countries’ development. ^7^

Among the diverse strategies to promote research among professors and students, ^8^ training, attracting, and retaining young researchers with aptitudes for research stand out, in addition to didactic techniques such as flipped classroom. ^9^ Thus, universities seek to train future researchers during the undergraduate programs, where research is one of the most important pillars in the education of future professionals, ^10^ particularly in the human medicine programs, and health sciences in general. This is intended to be replicated with greater demand and quality in the different postgraduate programs. ^1^^,^^9^

However, in developing countries and especially in Peru, universities do not fully comply with the function of training quality researchers. ^11^^,^^12^ This is manifested through the low level of medicine scientific production, compared to medical scientific production of countries such as the United States ^3^ or European countries. ^13^ This could be explained by the limited available economic or human resources for research, ^14^ a low level of research culture and deficient research training during the under- and postgraduate programs. ^15^

This situation leads research and scientific production to be a matter of interest and concern within the Peruvian scientific community and the Government. Thus, in 2014, the Peruvian University Law 30220 was passed, ^16^ which states that students should conduct research projects such as a thesis or a scientific article and this should be sustained and approved as a condition *sine qua non* to obtain their professional degree and finish satisfactorily the different postgraduate programs. This, in addition, is a basic condition of quality for the licensing of the different professional schools. ^1^

The courses that include research activity have a clear objective: to guide students in their scientific education. This situation requires that responsible professors have sufficient research experience expressed in a significant number of publications in indexed journals that certify scientific suitability and novelty. ^17^^,^^18^ Currently, there exists little available evidence that explores the scientific production of professors in Peru. In addition, this lack of evidence also includes scarce studies on factors associated with, particularly, the area of health sciences which reflects the current condition of the compliance of universities in terms of their scientific and training function, and offers truthful and pertinent information to implement programs and strategies that promote the scientific development of the country.

Taking into account what we stated above, this research’s objective was to estimate the association between professor’s academic, personal and work characteristics and their scientific production at the Universidad Norbert Wiener, Lima, Peru, in 2021, as a first step to identify those factors that contribute or limit the development of scientific production in a higher education institution in the capital of the country.

## Methods

### Design and population of the study

We undertook an observational, analytical, cross-sectional study. The population was made up of 663 professors of the Universidad Norbert Wiener during the first term of 2021, in Lima, Peru. We included both male and female professors that worked at the Universidad Norbert Wiener during the period of the study. Professors that did not want to participate or were not available to fill in the questionnaires during the period of the study were excluded.

The research instruments ^33^ were developed by the researchers and were applied using the Google Forms (Google, 2022) tool, for which the link to the survey was sent by e-mail to the study participants. The data provided by the participants were collected in Google drive (Google, 2022).

### Sampling type and size

A sample size was determined by proportional allocation according to the size of the school of origin, resulting in a total of 332 university teachers, after applying the statistical formulas. The sampling was simple random probabilistic, since there was a list of all the university teachers.

### Procedures

The information on the included participants in this study was collected through two questionnaires. The first was oriented towards the identification of academic, work, and personal characteristics. The second gathered information about the scientific production of the evaluated professors. Data collection was carried out via email, using the drive tool between the months of September and December 2020.

The data collection instruments were developed by the research team and validated through the Aiken’s V coefficient with the participation of ten expert methodologists and experts on the subject, who assessed clarity, objectivity, being up-to-date, organization, sufficiency, adequacy, coherence, methodology, and pertinence of the instruments’ content.

The reliability of the instrument was evaluated through a pilot test that included 30 professors from Norbert Wiener University, who were randomly selected and did not enter the study. The instruments were sent by email using Google drive (Google), then both instruments were subjected to the Cronbach's alpha and KR-20 reliability tests according to the type of variable evaluated. We obtained a Cronbach's alpha coefficient of 0.85 for the instrument that evaluates scientific production and 0.88 for academic factors. In addition, we obtained a KR-20 coefficient of 0.82 for personal factors and 0.90 for work factors. For which the SPSS version 25.0 (IBM Corp, 2017) (RRID:SCR_016479) was used, whose license is from Norbert Wiener University.

Reliability was assessed through a pilot test that included 30 professors of the Universidad Norbert Wiener. Both instruments were subjected to the Cronbach’s alpha and KR-20 tests according to the type of variable evaluated. We obtained a Cronbach’s alpha coefficient of 0.85 for the instrument that assesses scientific production and 0.88 for the one about academic factors. In addition, we obtained a KR-20 coefficient of 0.82 for the personal factors and 0.90 for the work factors the results of the instrument reliability test were adequate; therefore, no changes were made to the instruments. Data from the pilot study cannot be shared in this study, as they are being used for another study that will analyze the psychometric properties of the instruments.

### Bias

This study included professors of the Universidad Privada Norbert Wiener with socioeconomic characteristics that differ from the population of university teachers in Peru. However, this study is one of the first to evaluate professor’s scientific production and the possible associated factors, and it will serve as baseline for future studies with a wider scope.

Scientific production can be assessed in different ways that include journal type, H-index, language of publication, level of inter-institutional collaboration at a national as well as international level.

### Variables

*Outcome variable: Scientific production*

Professors’ scientific production was assessed as a dependent or outcome variable. This was defined as the number of publications (original article, review article, clinical case reports, books) in journals indexed in Scopus, Web of Science (WoS), Scielo, Latindex. In addition, the impact of publication through the H-index was reported. This information was obtained through the research instrument.

*Variables of exposure: Personal, academic and work characteristics*

Professors’ personal, academic, and work characteristics were evaluated as well as the postgraduate programs studied, the level of English comprehension, database management, reference management, statistical software management, subscription to scientific journals, memberships in scientific societies or groups, research competencies, or thesis advising.

Furthermore, work characteristics such as access to incentives, institutional support, infrastructure, and research funding were assessed, as well as administrative aspects such as employment category, contract type, and number of teaching and non-teaching hours.

In addition, we evaluated personal characteristics such as bio-social (gender, age, civil status, and children) and professional (profession, professional experience, teaching experience, or institutions where he/she worked) factors.

### Statistical analysis

The obtained data were collected in the Excel 2010 (Microsoft, 2010) (RRID:SCR_016137) software and were analyzed with the SPSS program version 25.0. The descriptive results of the categorical variables were shown through absolute and relative frequencies; while for quantitative variables, we calculated measures of central tendency and dispersion.

For assessing the relationship between each exposure and outcome variables, we used bivariate analysis and the Chi-squared test with their respective p-values. We considered p<0.05 statistically significant. We calculated prevalence ratios (PRs) and their respective confidence interval at 95% for each relationship.

Also, in order to evaluate the relationship between the variables of exposure and scientific production, a multiple logistic regression analysis was performed using the Stepwise method, where step by step we added the indicators that showed a value of p<0.05 to the previous bivariate analysis. All statistical analyzes were performed using SPSS version 25.0 software licensed by Norbert Wiener University.

### Ethical aspects

This study was carried out following the guidelines of the Declaration of Helsinki of 1964 and its subsequent modifications. In addition, the participants agreed to participate through written informed consent and we ensured the anonymity of the data obtained from each participant, so their integrity was not violated. This study was evaluated and approved by the Institutional Research Ethics Committee of the Norbert Wiener University. The approval number by the ethics committee was Exp. No 158-2020.

## Results

### Descriptive analysis of the scientific production in the study sample

The information obtained from a total of 322 professors, ^33^ allowed us to find that 59.6% (n=192) did not have publications in indexed journals and only 9.6% (n=31) had published six or more articles. By evaluating scientific production in different databases, we found that 13.4% (n=43) had a publication in Scopus, and 5.3% (n=17) had five or more. Also, 9.0% (n=29) had a publication in WoS and 3.1% (n=10) had four or more. In addition, 9.3% (n=30) had a publication in Scielo and 7.5% (n=24) had published four or more. We also found that 15.2% (n=49) published one or two papers in Latindex and 5% (n=16) had published five or more. On the other hand, 51.2% (n=165) did not have an H-index and 40.1% (n=129) did not know about this index. Moreover, 91.9% (n=296) had an H-index equal to 0, while 1.9% (n=6) had an H-index equal to or higher than 5 ( Table 1).

**Table 1.** Professors’ scientific production at the Universidad Norbert Wiener, 2022 (n=322).

	n	%
Publications in indexed journals		
No	192	59.6
Yes	130	40.4
Published articles		
None	190	59.0
1-3	80	24.8
4-5	21	6.5
≥6	31	9.6
Articles published in Open Access journals		
None	193	59.9
1-5	100	31.1
≥6	29	9.0
Articles published in Scopus		
None	251	78.0
1-2	43	13.4
3-4	11	3.4
≥5	17	5.3
Articles published in Web of Science (WoS)		
None	277	86.0
1	29	9.0
2-3	6	1.9
≥4	10	3.1
Articles published in Scielo		
None	256	79.5
1	30	9.3
2-3	12	3.7
≥4	24	7.5
Articles published in Latindex		
None	245	76.1
1-2	49	15.2
3-4	12	3.7
≥5	16	5.0
Has an H-index		
No	165	51.2
Yes	28	8.7
Does not know the H-index	129	40.1
Number of H-index		
0	296	91.9
1-2	13	4.0
3-4	7	2.2
≥5	6	1.9

### Descriptive and bivariate analysis by scientific production in the study sample

Of the 322 professors included in this study, 58.7% (n=189) belonged to the Faculty of Health Sciences; 6.8% (n=22), to Pharmacy and Biochemistry; 11.2% (n=36), to Engineering and Businesses; 9% (n=29), to Law and Political Sciences; and 14.3% (n=43), to the Postgraduate School.

Among the personal characteristics, we found that 59.3% (=191) were male and the median age was 48 (29-72 years old). We found that 16.8% of professors were registered in Renacyt (National Registry of Science, Technology and Technological Innovation), in which the most frequent category was Rostworowski III (3.4% n=11). Of the total, 98.4% stated that they had an interest in research and 50.3% (n=162) mentioned that they felt satisfaction due to research, but 62.7% (n=202) and 59.9% (n=193) indicated that economic aspects and workload, respectively, prevent them from conducting research. Through bivariate analysis, we could find an association between scientific production and belonging to the teaching staff of a postgraduate school (PR=2.05; 95% confidence interval: 1.38 to 3.04; p<0.001), being registered in DINA (PR=5.52; 95% confidence interval: 2.14 to 14.25; p<0.001) or in Renacyt (PR=2.39; 95% confidence interval: 1.92 to 2.98; p<0.001), and having received an award (RP=2.28; 95% confidence interval 1.81 to 2.88; p<0.001) or having being recognized for researching (PR=2.34; 95% confidence interval: 1.55 to 3.01; p<0.001) ( Table 2).

**Table 2.** Professors’ personal characteristics at the Universidad Norbert Wiener, 2022 (n=322).

Personal characteristics	n	%	Scientific production		Prevalence ratio [CI]	p-value *
			No (n=192) n (%)	Yes (n=130) n (%)		
Gender						
Male	191	59.3	117 (60.9)	74 (56.9)	1	
Female	131	40.7	75 (39.1)	56 (43.1)	1.10 [0.84-1.44]	0.470
Civil status						
Single	76	23.6	44 (22.9)	32 (24.6)	1	
Married	198	61.5	118 (61.5)	80 (61.5)	0.96 [0.70-1.31]	0.797
Domestic partner	21	6.5	15 (7.8)	6 (4.6)	0.68 [0.33-1.40]	0.296
Divorced	19	5.9	11 (5.7)	8 (6.1)	1.00 [0.55-1.80]	1.000
Widowed	8	2.5	4 (2.1)	4 (3.1)	1.19 [0.57-2.50]	0.650
Has children						
No	78	24.2	41 (21.4)	37 (28.5)	1	
Yes	244	75.8	151 (78.6)	93 (71.5)	0.80 [0.60-1.07]	0.130
Profession						
Non-related to Health Sciences	130	40.4	82 (42.7)	48 (36.9)	1	
Health Sciences	192	59.6	110 (57.3)	82 (63.1)	1.16 [0.88-1.53]	0.306
Number of institutions you are working for						
1	67	20.8	42 (21.9)	25 (19.2)	1	
2 or more	255	79.2	150 (78.1)	105 (80.8)	1.10 [0.78-1.56]	0.574
Time working at UNW **						
≤2 years	65	20.2	44 (22.9)	21 (16.1)	1	
>2 years	112	34.8	75 (39.1)	37 (28.5)	1.02 [0.66-1.59]	0.921
Does not respond	145	45.0	73 (38.0)	72 (55.4)	1.54 [1.04-2.27]	0.030
School in which you teach						
Non-related to Health Sciences	91	28.3	64 (33.3)	27 (20.8)	1	
Health Sciences	185	57.4	110 (57.3)	75 (57.7)	1.37 [0.95-1.96]	0.091
Postgraduate	46	14.3	18 (9.4)	28 (21.5)	2.05 [1.38-3.04]	**<0.001**
Registered in DINA ^†^						
No	48	14.9	44 (22.9)	4 (3.1)	1	
Yes	274	85.1	148 (77.1)	126 (96.9)	5.52 [2.14-14.25]	**<0.001**
Registered in Renacyt ^‡^						
No	273	84.8	182 (94.8)	91 (70.0)	1	
Yes	49	15.2	10 (5.2)	39 (30.0)	2.39 [1.92-2.98]	**<0.001**
Category						
Not registered in Renacyt	273	84.8	182 (94.8)	91 (70.0)	1	
María Rostworowski	26	8.1	4 (2.1)	22 (16.9)	2.54 [2.01-3.21]	**<0.001**
Carlos Monge	23	7.1	6 (3.1)	17 (13.1)	2.22 [1.65-2.98]	**<0.001**
Motivated to do research						
No	10	3.1	8 (4.2)	2 (1.5)	1	
Yes	312	96.9	184 (95.8)	128 (98.5)	2.05 [0.59-7.15]	0.259
How do you like to work when researching?						
In teams	234	72.7	130 (67.7)	104 (80.0)	1	
By myself	26	8.1	21 (10.9)	5 (3.8)	0.43 [0.19-0.96]	0.041
Indifferent	62	19.2	41 (21.4)	21 (16.2)	0.76 [0.52-1.11]	0.158
Role in the research team						
Leader of the project	116	36.0	55 (28.7)	61 (46.9)	1	
Member of the technical team	162	50.3	104 (54.2)	58 (44.6)	0.68 [0.52-0.89]	**0.005**
Other	44	13.7	33 (17.2)	11 (8.5)	0.48 [0.28-0.82]	**0.007**
Received a research award						
No	258	80.1	175 (91.1)	83 (63.9)	1	
Yes	64	19.9	17 (8.9)	47 (36.1)	2.28 [1.81-2.88]	**<0.001**
Was recognized for researching						
No	217	67.4	156 (81.2)	61 (46.9)	1	
Yes	105	32.6	36 (18.8)	69 (53.1)	2.34 [1.81-3.01]	**<0.001**
Is satisfied with research						
No	160	49.7	121 (63.0)	39 (30.0)	1	
Yes	162	50.3	71 (37.0)	91 (70.0)	2.30 [1.70-3.13]	**<0.001**
The economic situation prevents me from researching						
No	120	37.3	69 (35.9)	51 (39.2)	1	
Yes	202	62.7	123 (64.1)	79 (60.8)	0.92 [0.70-1.21]	0.547
Family load prevents me from researching						
No	223	69.2	133 (69.3)	90 (69.2)	1	
Yes	99	30.8	59 (30.7)	40 (30.8)	1.00 [0.75-1.34]	0.994
Work load prevents me from research						
No	129	40.1	78 (40.6)	51 (39.2)	1	
Yes	193	59.9	114 (59.4)	79 (60.8)	1.04 [0.79-1.36]	0.803

* P-value estimated through Chi-squared test, with a level of significance of p<0.05.

** Universidad Norbert Wiener.

^†^ National Directory of Researchers and Innovators.

^‡^ National Registry of Science, Technology and Technological Innovation.

Regarding the academic characteristics, we found that 99.4% (n=320) had a postgraduate degree and the most frequent was the master’s degree (81.4% n=262). In addition, 43.8% (n=141) had a basic level of English; while 14.3% (n=46) had an advanced level. According to what was declared by the study participants in the research survey. Likewise, Of the total, 85.7% (n=276) reported that they use a database for doing research. Also, 50.6% (n=163) stated that they used Google Scholar; 47.5% (n=153) used Scopus; 73.3% (n=253) used Scielo; 50.6% (n=163) used Pubmed; and 41.6% (n=134) used Medline. In addition, 54.7% (n=176) reported that they used reference managers; while 68.6% (n=221) used statistical software. Bivariate analysis showed that having a master’s degree was associated with not having scientific production (PR=0.65; 95% confidence interval: 1.49 to 0.85; p=0.002). Furthermore, scientific production was associated with having a doctoral degree (PR=2.14; 95% confidence interval: 1.60 to 2.85; p<0.001), having an intermediate level of English (PR=1.35; 95% confidence interval: 1.01 to 1.82; =0.045), having knowledge of bibliographic search in databases (PR=2.93; 95% confidence interval: 1.46 to 5.87; =0.002), and knowing how to use any statistical software (PR=2.05; 95% confidence interval: 1.39 to 3.03; p<0.001). In addition, belonging to a scientific society (PR=2.05; 95% confidence interval: 1.75 to 2.88; p<0.001) or to a research group (PR=3.67; 95% confidence interval: 2.66 to 5.07; p<0.001), and being a thesis advisor in a master’s program (PR=1.91; 95% confidence interval: 1.48 to 2.47; p<0.001) or in a doctoral program (PR=1.95; 95% confidence interval: 1.49 to 2.55; p<0.001) were also associated with a better level of scientific production ( Table 3).

**Table 3.** Professors’ academic characteristics and scientific production of the Universidad Norbert Wiener, 2022 (n=322).

Academic characteristics	n	%	Scientific production		Prevalence ratio [CI]	p-value *
			No (n=192) n (%)	Yes (n=130) n (%)		
Has specialty studies						
No	196	60.9	124 (64.6)	72 (55.4)	1	
Yes	126	39.1	68 (35.4)	58 (44.6)	1.25 [0.96-1.63]	0.094
Has a Master’s degree						
No	60	18.6	26 (13.5)	34 (26.1)	1	
Yes	262	81.4	166 (86.5)	96 (73.9)	0.65 [0.49-0.85]	**0.002**
Has a Doctorate degree						
No	171	53.1	126 (65.6)	45 (34.6)	1	
Yes	151	46.9	66 (34.4)	85 (65.4)	2.14 [1.60-2.85]	**<0.001**
Level of English comprehension						
Basic	141	43.8	93 (48.4)	48 (36.9)	1	
Intermediate	128	39.8	69 (35.9)	59 (45.4)	1.35 [1.01-1.82]	**0.045**
Advanced	46	14.3	27 (14.1)	19 (14.6)	1.21 [0.80-1.84]	0.361
None	7	2.2	3 (1.6)	4 (3.1)	1.68 [0.85-3.32]	0.137
Bibliographic search in databases						
No	46	14.3	39 (20.3)	7 (5.4)	1	
Yes	276	85.7	153 (79.7)	123 (94.6)	2.93 [1.46-5.87]	**0.002**
Bibliographic search in Google Scholar						
No	159	49.4	87 (45.3)	72 (55.4)	1	
Yes	163	50.6	105 (54.7)	58 (44.6)	0.79 [0.60-1.03]	0.078
Uses Scopus						
No	169	52.5	125 (65.1)	44 (33.9)	1	
Yes	153	47.5	67 (34.9)	86 (66.1)	2.16 [1.61-2.89]	**<0.001**
Uses Scielo						
No	86	26.7	55 (28.6)	31 (23.8)	1	
Yes	236	73.3	137 (71.4)	99 (76.2)	1.16 [0.85-1.60]	0.352
Uses Latindex						
No	247	76.7	155 (80.7)	92 (70.8)	1	
Yes	75	23.3	37 (19.3)	38 (29.2)	1.36 [1.03-1.79]	**0.029**
Uses EBSCO						
No	169	52.5	107 (55.7)	62 (47.7)	1	
Yes	153	47.5	85 (44.3)	68 (52.3)	1.21 [0.93-1.58]	0.158
Uses Pubmed						
No	159	49.4	107 (55.7)	52 (40.0)	1	
Yes	163	50.6	85 (44.3)	78 (60.0)	1.46 [1.11-1.93]	**0.007**
Uses Redalyc						
No	214	66.5	137 (71.4)	77 (59.2)	1	
Yes	108	33.5	55 (28.6)	53 (40.8)	1.36 [1.05-1.77]	**0.021**
Uses Medline						
No	188	58.4	127 (66.2)	61 (46.9)	1	
Yes	134	41.6	65 (33.8)	69 (53.1)	1.59 [1.22-2.07]	**0.001**
Uses reference managers						
No	81	25.2	57 (29.7)	24 (18.5)	1	
Yes	176	54.7	82 (42.7)	94 (72.3)	1.80 [1.25-2.59]	**0.001**
Does not know them	65	20.2	53 (27.6)	12 (9.2)	0.62 [0.34-1.15]	0.130
Uses Mendeley						
No	199	61.8	141 (73.4)	58 (44.6)	1	
Yes	123	38.2	51 (26.6)	72 (55.4)	2.01 [1.54-2.61]	**<0.001**
Uses Zotero						
No	258	80.1	165 (85.9)	93 (71.5)	1	
Yes	64	19.9	27 (14.1)	37 (28.5)	1.60 [1.23-2.09]	**<0.001**
Uses EndNote						
No	300	93.2	183 (95.3)	117 (90.0)	1	
Yes	22	6.8	9 (4.7)	13 (10.0)	1.52 [1.04-2.21]	**0.030**
Uses other reference manager						
No	280	87.0	164 (85.4)	116 (89.2)	1	
Yes	42	13.0	28 (14.6)	14 (10.8)	0.80 [0.51-1.26]	0.344
Manages any statistical software						
No	94	29.2	72 (37.5)	22 (16.9)	1	
Yes	221	68.6	115 (59.9)	106 (81.5)	2.05 [1.39-3.03]	**<0.001**
Does not know them	7	2.2	5 (2.6)	2 (1.5)	1.22 [0.36-4.17]	0.750
Uses Stata						
No	280	87.0	178 (92.7)	102 (78.5)	1	
Yes	42	13.0	14 (7.3)	28 (21.5)	1.83 [1.40-2.38]	**<0.001**
Uses SPSS						
No	54	16.8	37 (19.3)	17 (13.1)	1	
Yes	268	83.2	155 (80.7)	113 (86.9)	1.34 [0.88-2.04]	0.171
Uses Atlas ti						
No	290	90.1	181 (94.3)	109 (83.9)	1	
Yes	32	9.9	11 (5.7)	21 (16.1)	1.75 [1.30-2.34]	**<0.001**
Uses Minitab						
No	299	92.9	179 (93.2)	120 (92.3)	1	
Yes	23	7.1	13 (6.8)	10 (7.7)	1.08 [0.67-1.76]	0.747
Uses other software						
No	252	78.3	153 (79.7)	99 (76.2)	1	
Yes	70	21.7	39 (20.3)	31 (23.8)	1.13 [0.83-1.53]	0.441
Who performs the statistical analysis?						
You do it	131	40.7	65 (33.8)	66 (50.8)	1	
Another researcher	110	34.2	61 (31.8)	49 (37.7)	0.88 [0.67-1.16]	0.370
Others, by hiring them	81	25.2	66 (34.4)	15 (11.5)	0.37 [0.23-0.60]	**<0.001**
Subscribed to any scientific journal						
No	247	76.7	170 (88.5)	77 (59.2)	1	
Yes	75	23.3	22 (11.5)	53 (40.8)	2.27 [1.79-2.87]	**<0.001**
Member of any Scientific Society						
No	227	70.5	160 (83.3)	67 (51.5)	1	
Yes	95	29.5	32 (16.7)	63 (48.5)	2.25 [1.75-2.88]	**<0.001**
Society you belong to						
None	227	70.5	160 (83.3)	67 (51.5)	1	
Medicine and specialties	25	7.8	10 (5.2)	15 (11.5)	2.03 [1.39-2.97]	**<0.001**
Society/association of stomatology or dentistry	21	6.5	10 (5.2)	11 (8.5)	1.77 [1.13-2.80]	**0.014**
Related to science, technology or environment	14	4.3	1 (0.5)	13 (10.0)	3.15 [2.45-4.03]	**<0.001**
Others	35	10.9	11 (5.7)	24 (18.5)	2.32 [1.72-3.14]	**<0.001**
Belongs to any research group						
No	182	56.5	148 (77.1)	34 (26.2)	1	
Yes	140	43.5	44 (22.9)	96 (73.9)	3.67 [2.66-5.07]	**<0.001**
Leader of any research group						
No	253	78.6	176 (91.7)	77 (59.2)	1	
Yes	69	21.4	16 (8.3)	53 (40.8)	2.52 [2.01-3.17]	**<0.001**
Participated in research in the last year						
No	134	41.6	114 (59.4)	20 (15.4)	1	
Yes	188	58.4	78 (40.6)	110 (84.6)	3.92 [2.57-5.98]	**<0.001**
Number of research studies in which you have participated						
None	133	41.3	115 (59.9)	18 (13.9)	1	
1	75	23.3	48 (25.0)	27 (20.8)	2.66 [1.57-4.50]	**<0.001**
2	58	18.1	18 (9.4)	40 (30.8)	5.10 [3.21-8.10]	**<0.001**
3	26	8.1	6 (3.1)	20 (15.4)	5.68 [3.52-9.18]	**<0.001**
4 or more	30	9.3	5 (2.6)	25 (19.2)	6.16 [3.89-9.74]	**<0.001**
Attended methodology training sessions						
No	54	16.8	41 (21.4)	13 (10.0)	1	
Yes	268	83.2	151 (78.6)	117 (90.0)	1.81 [1.11-2.97]	**0.018**
Has been a thesis advisor						
No	75	23.3	60 (31.2)	15 (11.5)	1	
Yes	247	76.7	132 (68.8)	115 (88.5)	2.33 [1.45-3.73]	**<0.001**
Bachelor’s advisor						
No	151	46.9	85 (44.3)	66 (50.8)	1	
Yes	171	53.1	107 (55.7)	64 (49.2)	0.86 [0.66-1.12]	0.252
Second specialty advisor						
No	255	79.2	153 (79.7)	102 (78.5)	1	
Yes	67	20.8	39 (20.3)	28 (21.5)	1.05 [0.76-1.44]	0.789
License program advisor						
No	163	50.6	100 (52.1)	63 (48.5)	1	
Yes	159	49.4	92 (47.9)	67 (51.5)	1.09 [0.84-1.42]	0.524
Master’s advisor						
No	209	64.9	145 (75.5)	64 (49.2)	1	
Yes	113	35.1	47 (24.5)	66 (50.8)	1.91 [1.48-2.47]	**<0.001**
Doctoral advisor						
No	293	91.0	184 (95.8)	109 (83.8)	1	
Yes	29	9.0	8 (4.2)	21 (16.2)	1.95 [1.49-2.55]	**<0.001**

* P-value estimated through the Chi-squared test, with a level of significance of p<0.05.

In regard to work characteristics, only 5.9% (n=19) were professors with a permanent appointment and 76.7% (n=247) did not have a regulated teaching category. Also, 73.3% (n=236) indicated that the university did not grant them time for research and only 11.5% (n=37) dedicated 10 or more hours a week to research-related activities. In addition, 87.9% (n=283) had not received any funding for conducting research; 90.7% (n=292) had not received an incentive to publish; and 727% (n=234) had not received any support in the management of research projects. Bivariate analysis showed that scientific production was associated with having facilities to conduct research (PR=148; 95% confidence interval: 1.12 to 1.97; p=0.006) or having been trained in research (PR=1.55; 95% confidence interval: 1.12 to 2.13; p=0.008); and having received funding (PR=1.99; 95% confidence interval: 1.55 to 2.56; p<0.001) or a research incentive (PR=1.98; 95% confidence interval: 1.52 to 2.58; p<0.001) ( Table 4).

**Table 4.** Professors’ work characteristics and their scientific production at the Universidad Norbert Wiener, 2022 (n=322).

Work factors	n	%	Scientific production		Prevalence ratio [CI]	p-value *
			No (n=192) n (%)	Yes (n=130) n (%)		
Facilities to conduct research						
No	147	46.6	100 (52.1)	47 (36.2)	1	
Yes	175	54.4	92 (47.9)	83 (63.8)	1.48 [1.12-1.97]	**0.006**
Has hours a week granted to do research						
No	236	73.3	144 (75.0)	92 (70.8)	1	
Yes	86	29.7	48 (25.0)	38 (29.2)	1.13 [0.85-1.51]	0.392
Research hours a week						
None	176	54.7	109 (56.8)	67 (51.5)	1	
1-4	60	18.6	41 (21.3)	19 (14.6)	0.83 [0.55-1.26]	0.387
5-9	49	15.2	22 (11.5)	27 (20.8)	1.45 [1.06-1.98]	**0.022**
≥10	37	11.5	20 (10.4)	17 (13.1)	1.21 [0.81-1.80]	0.354
Chief encourages you to do research						
No	137	42.6	95 (49.5)	42 (32.3)	1	
Yes	185	57.4	97 (50.5)	88 (67.7)	1.55 [1.16-2.08]	**0.003**
Over the last year, have you received training?						
No	111	34.5	78 (40.6)	33 (25.4)	1	
Yes	211	65.5	114 (59.4)	97 (74.6)	1.55 [1.12-2.13]	**0.008**
Received research funding						
No	283	87.9	181 (94.3)	102 (78.5)	1	
Yes	39	12.1	11 (5.7)	28 (24.5)	1.99 [1.55-2.56]	**<0.001**
Received an incentive to publish						
No	292	90.7	184 (95.8)	108 (83.1)	1	
Yes	30	9.3	8 (4.2)	22 (16.9)	1.98 [1.52-2.58]	**<0.001**
Has a physical infrastructure						
No	70	21.7	41 (21.4)	29 (22.3)	1	
Yes	252	78.3	151 (78.7)	101 (77.7)	0.97 [0.70-1.33]	0.838
Has technological resources						
No	55	17.1	27 (14.1)	28 (21.5)	1	
Yes	267	82.9	165 (85.9)	102 (78.5)	0.75 [0.56-1.01]	0.062
Has bibliographic resources						
No	55	17.1	28 (14.6)	27 (20.8)	1	
Yes	267	82.9	164 (85.4)	103 (79.2)	0.79 [0.58-1.07]	0.127
Received support in the management of projects						
No	234	72.7	156 (81.2)	78 (60.0)	1	
Yes	88	27.3	36 (18.8)	52 (40.0)	1.77 [1.38-2.28]	**<0.001**
Facilities for external funding						
No	286	88.8	181 (94.3)	105 (88.8)	1	
Yes	36	11.2	11 (5.7)	25 (19.2)	1.89 [1.45-2.47]	**<0.001**
Promotes national internships						
No	210	65.2	132 (68.8)	78 (60.0)	1	
Yes	112	34.8	60 (31.2)	52 (40.0)	1.25 [0.96-1.63]	0.100
Promotes international internships						
No	209	64.9	129 (67.2)	80 (61.5)	1	
Yes	113	35.1	63 (32.8)	50 (38.5)	1.16 [0.88-1.51]	0.292
Provides support with translation of articles						
No	207	64.3	136 (70.8)	71 (54.6)	1	
Yes	115	35.7	56 (29.2)	59 (45.4)	1.50 [1.15-1.94]	**0.002**
Research studies according to institutional research lines						
No	30	9.3	22 (11.5)	8 (6.1)	1	
Yes	292	90.7	170 (88.5)	122 (93.9)	1.57 [0.85-2.88]	0.149
Has an Ethics Committee for research						
No	21	6.5	15 (7.8)	6 (4.6)	1	
Yes	301	93.5	177 (92.4)	124 (95.4)	1.44 [0.72-0.88]	0.299
Professor’s category						
Is not categorized	247	76.7	149 (77.6)	98 (75.4)	1	
Associate	33	10.2	19 (9.9)	14 (10.8)	1.07 [0.70-1.64]	0.758
Main/Professor	12	3.7	4 (2.1)	8 (6.1)	1.68 [1.09-2.58]	0.180
Assistant	30	9.3	20 (10.4)	10 (7.7)	0.84 [0.50-1.43]	0.519
Work contract type						
Permanent appointment	19	5.9	9 (4.7)	10 (7.7)	1	
Full-time	80	24.8	41 (21.3)	39 (30.0)	0.93 [0.57-1.50]	0.756
Part-time	223	69.3	142 (74.0)	81 (62.3)	0.69 [0.44-1.10]	0.115
Teaching hours a week						
None	33	10.3	21 (10.9)	12 (9.2)	1	
3-12	97	30.1	51 (26.6)	46 (35.4)	1.30 [0.79-2.15]	0.296
13-20	132	41.0	83 (43.2)	49 (37.7)	1.02 [0.62-1.69]	0.936
≥21	60	18.6	37 (19.3)	23 (17.7)	1.05 [0.61-1.84]	0.852
Non-teaching hours a week						
None	197	61.2	124 (64.6)	73 (56.1)	1	
1-17	34	10.6	19 (9.9)	15 (11.5)	1.19 [0.78-1.81]	0.416
18-35	60	18.6	32 (16.7)	28 (21.5)	1.26 [0.91-1.75]	0.166
≥36	31	9.6	17 (8.9)	14 (10.8)	1.22 [0.79-1.87]	0.366

* P-value estimated through the Chi-squared test, with a level of significance of p<0.05.

*Multivariate analysis by scientific production in the study sample*

Through multiple logistic regression analysis, using the Stepwise method, it was found that the main factors associated with scientific production were being registered in Renacyt (adjusted prevalence ratio (aPR)=5.52; 95% confidence interval: 2.14 to 4.23; p<0.001), having a doctoral degree (aPR=2.45; 95% confidence interval: 1.60 to 3.85; p<0.001), and having being a thesis advisor (aPR=3.83; 95% confidence interval: 1.45 to 5.66; p<0.001) ( Table 5).

**Table 5.** Logistic regression analysis of the professors’ personal, academic, and work characteristics and their scientific production at the Universidad Norbert Wiener, 2022 (n=322).

Characteristics	Adjusted Prevalence ratios (aPR)	Confidence interval (CI)	p-value *
Being registered in Renacyt **	5.52	[2.14-4.23]	**<0.001**
Having a doctoral degree	2.45	[1.60-3.85]	**<0.001**
Having been a thesis advisor	3.83	[1.45-5.66]	**<0.001**
Having facilities for research activities at work	1.58	[1.12-2.47]	**0.006**
Having received training by the university	1.99	[1.55-2.56]	**<0.001**

* P-value estimated through multiple logistic regression using the Stepwise method, with a level of significance of p<0.05.

** National Registry of Science, Technology and Technological Innovation.

## Discussion

This study’s objective was to estimate the association between university teachers’ scientific production and their personal, academic, and work characteristics to give basic data that enable the implementation or modification of strategies that encourage professors to conduct research.

Scientific research is an essential and mandatory activity of universities, which, according to the legal Peruvian university framework, ^16^ “are academic communities oriented towards research and teaching”. In addition to being an indicator for national and international licensing and accreditation processes, scientific production is the best way to measure research competencies in professors. ^18^ This is why, it is indispensable for university teachers to be sufficiently trained, as they are the first contact that students will have with research ^19^ and professors are who encourage them to do research in an adequate way. ^17^

However, this study found that barely 40% of professors have published a scientific article in indexed journals, and only 9.6% had more than six publications in those journals. In addition, 22.1% had, at least, a publication in Scopus and 20.5% in Scielo. These findings are similar to what was reported by previous studies such as the one by Alarcón-Ruiz, ^20^ who found out, in 2018, that 26% of the assessed professors had a publication in Scopus over the last five years, while only 5% had a publication in the same database over the last two years. Chachaima-Mar J ^17^ also reported similar percentages as this author found that only 14.6% of the sample had published in Scielo and 5.9%, in Scopus over the last three years, and LILACS was the most commonly used database (34.4%). Despite these similarities, it is possible to note that in our research we found a better percentage of professors with at least one published article in indexed journals. This evidences that this indicator has improved and that, if it continues in the subsequent years, it is possible to reverse the current situation in regard to professors’ scientific production in Peru.

In addition, a factor related to the number of publications is the H-index, which measures the impact of those publications. In this study, we found that only 8,7% of professors had an H-index, which coincides with what was reported in another study. ^21^ These findings could be the reflection of the low level of culture of publication in the university community, despite the fact that conducting research, many times do not end up in publication due to the little initiative, training or funding, among others. ^22^

Definitely, having teaching staff that do not publish indicates that these professors are not trained to teach research, for which it is crucial to study the factors involving the lack of scientific production of university teachers. Thus, this study found that receiving awards, incentives or funding for researching favors scientific production, as reported in other studies. ^14^^,^^23^ This might be explained by the great economic expenses involving the process of developing a research project, its execution, and publication, which evidence that adequate and well-oriented funding of research activities would greatly contribute to the improvement of publication indexes in professors of Peruvian universities.

However, we have to take into account that, although economic incentives motivate professors and increase publications in universities, it has been demonstrated that they can decrease the quality of the published articles, which increases plagiarism, salami sliced or redundant publications and generates authorship problems. ^24^ This could be solved if incentives are directed to payment for publications in journals with a high impact factor, and with the implementation of surveillance to guarantee scientific integrity.

In addition, in our study, personal characteristics such as gender or civil status were not associated with scientific production, which coincides with what was reported with evidence. ^25^^,^^26^ It is important to mention that significant differences have been reported in terms of female and male scientific production, highlighting the low level of representativity of female scientists in the published articles. ^27^ This contrast could be due to the methodological or sample characteristics’ differences between the present study and the cited studies, although this found difference serves as the basis for questioning why there is still a low level of women’s scientific production. This led us to refocus on the existing gender gaps in the academic and scientific community. ^28^ However, this is different when comparing women’s scientific production by academic areas as reported by Valdespinto-Aberti, ^29^ who found female predominance in pediatrics publications, and by Holman *et al.,* ^27^ who reported the predominance of female authors in nursing and obstetrician areas.

In this study we did not find an association between time working as a professor and scientific production, which is similar to what was reported by another study in 2018. ^26^ This could be due to the fact that probably professionals’ research practice is, in many cases, related to pre-professional training. Therefore, independently from the time a professional is practicing as a professor, it will be more complicated that they conduct research if they have not been motivated during their university education. In addition, a study found that younger professors were the ones with the most of scientific production. ^25^ This could probably be due to the fact that this group has a higher level of familiarity with technological resources currently available and which are used for research and by journals, which evidences that the time of teaching practice is not a relevant factor in scientific production.

Moreover, we found that doctoral education is associated with a higher frequency of publications. This finding has already been reported in other studies, in which it was evidenced that the higher academic degree, the higher level of participation in research, ^28^^,^^29^ which is related to the objective of doctoral education: to produce new knowledge from research. This shows the importance of scientific preparation in university teachers in order to improve indicators of scientific production. To this end, it is crucial to implement programs and strategies for the promotion and the following of research practices in professors. ^30^^,^^31^

This study had some limitations: 1) the population included was comprised of professors of the Universidad Norbert Wiener, which could not be representative of the totality of the country in regard to socioeconomic characteristics; 2) Assessing scientific production has multiple nuances, which goes beyond the type of journal in which the professor publishes and the H-index, including also the published language and the level of inter-institutional collaboration nationally as well as intentionally; 3) Due to the fact that the instrument was created specifically for this study, it is not possible to directly compare the results with other studies that used instruments with different structure and content. Despite these limitations, this study is one of the first to focus the attention on specific population and, because of that, can serve as a basis for subsequent studies with a wider scope and for decision-making in regard to strategies, programs, and policies that seek to promote and strengthen research in university teachers.

## Conclusions

To conclude, scientific production is related to being registered in Renacyt, having a doctoral degree, having been a thesis advisor, having facilities to conduct research activities at the workplace, and having been trained in research by the university. These findings are consistent with what was described in the literature and let us know the current situation of professors. Scientific production is low, which has an impact on the educational environment, since professors are not participating in one of the main functions of the university and are not contributing to the development of Peru. Therefore, it is necessary to reinforce the universities’ evaluation systems with respect to university quality standards that allow a better monitoring of professors’ research practice. In addition, wider scope studies should be conducted to know the current situation of professors’ scientific production and its associated factors at a national level. This will enable the implementation of strategies that promote research in professors in order to improve scientific production indicators.

## Data availability

### Underlying data

Zenodo: Factors associated with scientific production of professors working at a private university in Peru: An analytical cross-sectional study, https://doi.org/10.5281/zenodo.7067303. ^32^

This project contains the following underlying data:

- Factors associated with scientific production of professors working at a private university in Peru An analytical cross-sectional study.xlsx (All raw data collected).

### Extended data

Data from the pilot study cannot be shared in this study, as they are being used for another study that will analyze the psychometric properties of the instruments.

Zenodo: Instruments-Factors associated with scientific production of professors working at a private university in Peru: An analytical cross-sectional study, https://doi.org/10.5281/zenodo.7091241. ^33^

- This project contains the following extended data: Instrument.pdf (Combined questionnaires used in study).

Data are available under the terms of the Creative Commons Attribution 4.0 International license (CC-BY 4.0).

## Consent

Written informed consent for publication of the participants details was obtained from the participants. The approval number by the ethics committee was Exp. No 158-2020.
