# Supplementary material for: Factors associated with scientific production of professors working at a private university in Peru: An analytical cross-sectional study
Source: F1000Res. 2022 Oct 26;11:1219. [Version 1] doi: 10.12688/f1000research.126143.1 (PMC11074689; doi:10.12688/f1000research.126143.1)
Supplement: Peer review history

# Reviewer report: Reviewer response for version 1

**DOI:** 10.5256/f1000research.138523.r266036

**Published:** 2024-05-24

**Authors:** Boris Salazar

**Affiliations:** 1 Universidad del Valle, Cali, Colombia

Peer Review of Oriana Rivera-Lozada, ISABEL CRISTINA RIVERA-LOZADA2, and Cesar Antonio Bonilla-Asalde, “Factors associated with scientific production of professors

 working at a private university in Peru: An analytical cross-sectional study”.

 The article under review is an attempt to explore the statistical association between the academic, personal and work characteristics of a sample of 332 university teachers from a private university in Peru, and their scientific production. The authors are adamant about their motivations: they know how far from the world’s top-tier scientists’ outputs is the Peruvian scientific production –measured by the number of publications in peer-reviewed journals of the sample of teachers under study, and by their H-indexes— and how a huge share of that gap is related to the absence, in Peruvian universities, of serious and well-funded research training programs for young motivated undergraduate students. Would-be or future university teachers that did not learn from early youth the secrets of scientific research will be in no position to contribute to the training of an increasing number of future scientists in their countries.

 As the authors avoided the difficulties of causal analysis, they concentrated their efforts in finding the most credible data to measure what they tagged as the “dependent or outcome variable”, that is, the scientific production of the sample of 332 university teachers from the Universidad Norbert Weiner. They had on their side the powerful indexes, processed and provided by different publishing and research organizations, that put into numbers the amount and impact of individual researchers’ scientific publications.

 What they denominated as “variables of exposure” were divided in two parts: those belonging to the individuals and those related to the institutional, academic, professional, occupational and working arrangements provided by the university and the Peruvian higher educational system. Regrettably the interactions between these two types of variables were not directly addressed by the authors.

 In the first part the authors included “the level of English comprehension, database management, reference management, statistical software management, subscription to scientific journals, memberships in scientific societies or groups, research competencies, or thesis advising (p. 4).” These characteristics read as the research endowment of every university teacher:  his or her capacities, abilities and competencies for conducting research. Are those endowments inherent to each individual, and to his or her decisions, or are they the result of paths forced upon them by forces beyond their control, including the incentives and institutional arrangements of Peruvian universities and university system?

 The second part correspond to what they classified as the “work characteristics”: “access to incentives, institutional support, infrastructure, and research funding were assessed, as well as administrative aspects such as employment category, contract type, and number of teaching and non-teaching hours (idem)”. These, of course, reflect the institutional arrangements of both the University of reference and the Peruvian Higher Education system.

 A clear correlation between “the economic situation prevents me from research”, “family load prevents me for research”, heavy “work load prevents me from research” and not “receiving any funding for research”, on the one hand, and *no* scientific production was clearly detected, with “87.9% not having received any funds for conducting research” (p. 8). 

 They also found the 59.6% of the university teachers under study did not have any publication in indexed journals. This finding goes in line with an even more telling discovery: 40.1% of the teachers surveyed did not even know of the existence of the H-index! It is difficult to find a shaper signal of the separate existence of the university teachers surveyed in two different, unrelated worlds: one in which the H-index gives each one of them a place in a hierarchy, and another one in which that index does not even exist because it does not belong into the real lives of those university teachers.

 Regretfully, as I said before, these findings and the interactions between the institutional rules of individual universities and of the Peruvian Higher Education system and the personal traits of the university teachers were not fully exploited in the discussion section of the paper.

 There is, however, a logistic regression of the professors’ personal academic and work characteristics and their scientific production (p.14) that in some way deals with the interaction issue I raised above. Because they estimated a logistic regression they found what type of characteristics, personal and work related, were *probabilistically associated* with having a higher scientific production output. It comes as no surprise that the teachers registered in Renacyt, having a doctoral degree, having been a thesis advisor, having facilities for research activities at work and having received training by the universities, are the ones with the highest scientific production in the sample of university teachers from the Norbert Weiner University.

 It is pretty obvious that this group of teachers belong into the “fat tail” of the distribution of probability of the scientific production of the sample of reference: they are the few chosen, that with a smaller probability, have a large scientific production and an H-index larger that 6. Most of the remaining teachers in the sample have a large probability of having 0 or a very small scientific production. This is not a strange finding, only related to the Norbert Weiner’s teachers. On the contrary, it does reflect the world distribution of probability of the scientific production of university teachers. Only an elite of university teachers concentrates most of the world’s scientific production.

 My humble recommendation is that the authors give some extra lines to the discussion of the interactions between university teachers’ personal and work traits and the institutional arrangements of the Norbert Weiner University and of the Peruvian University at large. A more nuanced discussion of the findings in page 14 will clearly improve the interpretation and discussion powers of the article.

 It goes without saying that I recommend this article under review.

Is the work clearly and accurately presented and does it cite the current literature?

Yes

If applicable, is the statistical analysis and its interpretation appropriate?

Yes

Are all the source data underlying the results available to ensure full reproducibility?

Yes

Is the study design appropriate and is the work technically sound?

Yes

Are the conclusions drawn adequately supported by the results?

Yes

Are sufficient details of methods and analysis provided to allow replication by others?

Yes

Reviewer Expertise:

NA

I confirm that I have read this submission and believe that I have an appropriate level of expertise to confirm that it is of an acceptable scientific standard.

# Reviewer report: Reviewer response for version 1

**DOI:** 10.5256/f1000research.138523.r266037

**Published:** 2024-05-06

**Authors:** Jose Antonio Grandez Urbina

**Affiliations:** 1 Universidad Continental, Lima, Peru

The article investigates the factors influencing scientific production among professors at Universidad Norbert Wiener in Lima, Peru, in 2021. It discusses the importance of scientific production for the development of universities and countries, particularly in the context of the Peruvian scientific community's concerns about low scientific output. The study aims to identify factors that contribute to or limit scientific production in higher education institutions.

 The research employs an observational, analytical, cross-sectional study design with a sample size of 322 university professors. Data collection involved questionnaires addressing academic, personal, and work characteristics, as well as scientific production. Statistical analysis included descriptive analysis, bivariate analysis, and multiple logistic regression.

 Key findings include:

- Only 40% of professors had published scientific articles in indexed journals.
- Factors positively associated with scientific production include being registered in Renacyt, holding a doctoral degree, and being a thesis advisor.
- Personal, academic, and work characteristics such as gender, civil status, time working as a professor, and having a master's degree did not significantly influence scientific production.
- Challenges identified include limited funding, lack of incentives, and workload hindering research activities.

Is the work clearly and accurately presented and does it cite the current literature?

Yes

If applicable, is the statistical analysis and its interpretation appropriate?

Yes

Are all the source data underlying the results available to ensure full reproducibility?

Yes

Is the study design appropriate and is the work technically sound?

Yes

Are the conclusions drawn adequately supported by the results?

Yes

Are sufficient details of methods and analysis provided to allow replication by others?

Yes

Reviewer Expertise:

Health Sciences and Regenerative Medicine

I confirm that I have read this submission and believe that I have an appropriate level of expertise to confirm that it is of an acceptable scientific standard.

# Reviewer report: Reviewer response for version 1

**DOI:** 10.5256/f1000research.138523.r162954

**Published:** 2023-04-27

**Authors:** Salomon Huancahuire-Vega

**Affiliations:** 1 Universidad Peruana Union, Lima District, Lima Region, Peru

This is an interesting study that measures the association of scientific production with certain personal, academic, and work characteristics of university professors. The study was carried out with professors from a private university in Lima. The manuscript is well organized and written. However, there are some issues that need to be clarified:

 In the Methods section, design and population of the study subsection, it is mentioned that the study was carried out during the first term of 2021. However, in the Procedures subsection, they mention between September and December 2020. Correct the information.

 The last two paragraphs of the Procedures section have the same information, that is, they are duplicated. Correct.

 About the variable Scientific production. Were editor-letters and editorials not considered publications?

 If you have access to the databases, why didn't the researchers collect the scientific production data of the university professors directly from the databases? This form would have helped to reduce some memory bias of the participants when completing the questionnaires on scientific production.

Is the work clearly and accurately presented and does it cite the current literature?

Yes

If applicable, is the statistical analysis and its interpretation appropriate?

Yes

Are all the source data underlying the results available to ensure full reproducibility?

Partly

Is the study design appropriate and is the work technically sound?

Yes

Are the conclusions drawn adequately supported by the results?

Yes

Are sufficient details of methods and analysis provided to allow replication by others?

Yes

Reviewer Expertise:

Medical education and Public health

I confirm that I have read this submission and believe that I have an appropriate level of expertise to confirm that it is of an acceptable scientific standard.
